# Association of the Tyrosine/Nitrotyrosine pathway with death or ICU admission within 30 days for patients with community acquired pneumonia

**DOI:** 10.1186/s12879-018-3335-y

**Published:** 2018-08-24

**Authors:** Thomas Baumgartner, Giedré Zurauskaité, Yannick Wirz, Marc Meier, Christian Steuer, Luca Bernasconi, Andreas Huber, Mirjam Christ-Crain, Christoph Henzen, Claus Hoess, Robert Thomann, Werner Zimmerli, Beat Mueller, Philipp Schuetz

**Affiliations:** 1 Division of Endocrinology, Diabetology and Metabolism, Kantonsspital Aarau, Medical University Department, Aarau, Switzerland; 2 Department of Laboratory Medicine Kantonsspital Aarau, Aarau, Switzerland; 3 Endocrinology, Diabetology and Metabolism, University Hospital Basel, Basel, Switzerland; 4 Department of Internal Medicine, Kantonsspital Luzern, Lucerne, Switzerland; 5 Department of Internal Medicine, Kantonsspital Münsterlingen, Münsterlingen, Switzerland; 6 Department of Internal Medicine, Bürgerspital Solothurn, Solothurn, Switzerland; 7 Department of Internal Medicine, Kantonsspital Liestal, Liestal, Switzerland

## Abstract

**Background:**

Oxidative stress is a modifiable risk-factor in infection causing damage to human cells. As an adaptive response, cells catabolize Tyrosine to 3-Nitrotyrosine (Tyr-NO2) by nitrosylation. We investigated whether a more efficient reduction in oxidative stress, mirrored by a lowering of Tyrosine, and an increase in Tyr-NO2 and the Tyrosine/Tyr-NO2 ratio was associated with better clinical outcomes in patients with community-acquired pneumonia (CAP).

**Methods:**

We measured Tyrosine and Tyr-NO2 in CAP patients from a previous randomized Swiss multicenter trial. The primary endpoint was adverse outcome defined as death or ICU admission within 30-days; the secondary endpoint was 6-year mortality.

**Results:**

Of 278 included CAP patients, 10.4% experienced an adverse outcome within 30 days and 45.0% died within 6 years. After adjusting for the pneumonia Severity Index [PSI], BMI and comorbidities, Tyrosine nitrosylation was associated with a lower risk for short-term adverse outcome and an adjusted OR of 0.44 (95% CI 0.20 to 0.96, *p* = 0.039) for Tyr-NO2 and 0.98 (95% CI 0.98 to 0.99, *p* = 0.043) for the Tyrosine/Tyr-NO2 ratio. There were no significant associations for long-term mortality over six-years for Tyr-NO2 levels (adjusted hazard ratio 0.81, 95% CI 0.60 to 1.11, *p* = 0.181) and Tyrosine/Tyr-NO2 ratio (adjusted hazard ratio 1.00, 95% CI 0.99 to 1.00, *p* = 0.216).

**Conclusions:**

Tyrosine nitrosylation in our cohort was associated with better clinical outcomes of CAP patients at short-term, but not at long term. Whether therapeutic modulation of the Tyrosine/Tyr-NO2 pathway has beneficial effects should be evaluated in future studies.

**Trial registration:**

ISRCTN95122877. Registered 31 July 2006.

## Background

**Fig. 1:**
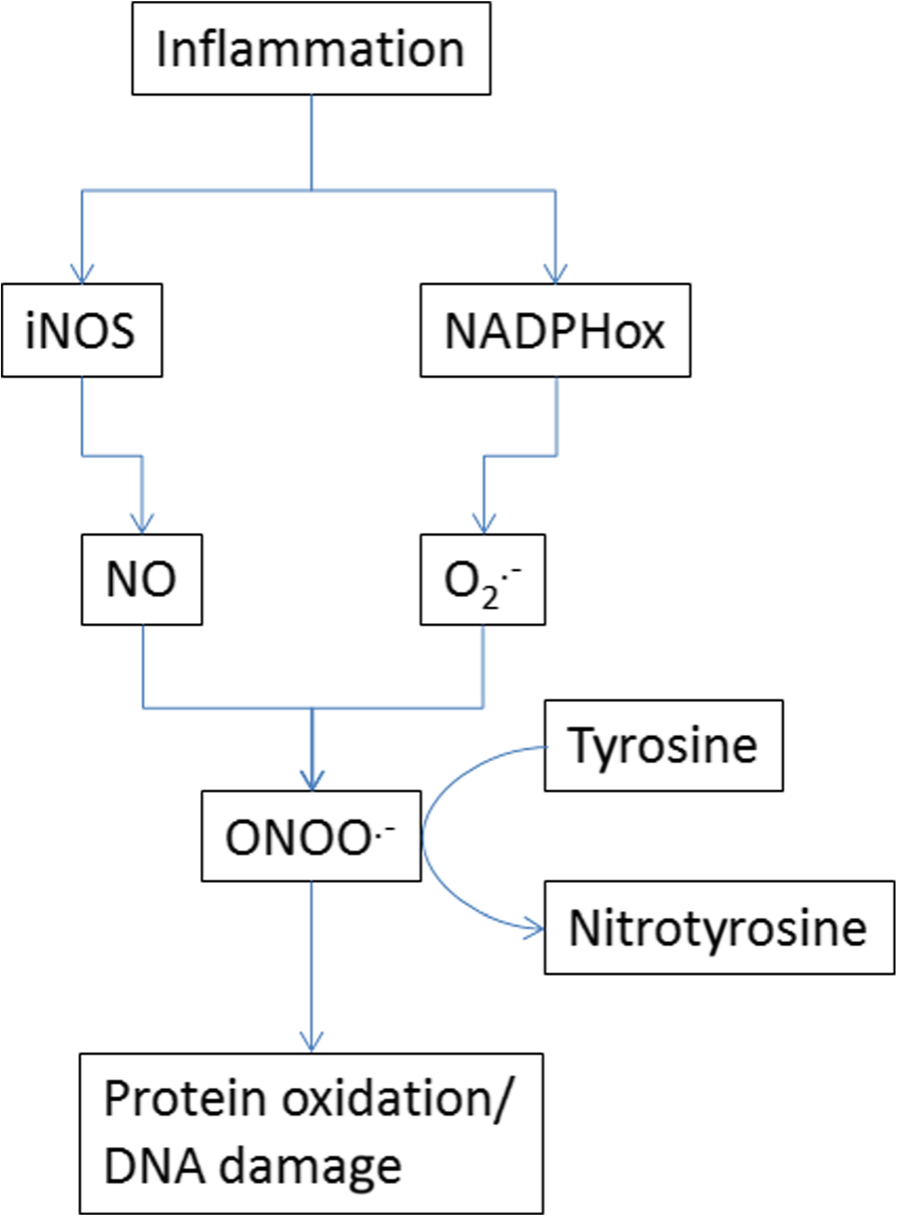
Abbreviated physiological biochemistry of the Tyrosine-Peroxynitrite-Tyr-NO2 pathway. Legend: iNOS, inducible Nitric-Oxide synthetase; NADPHox, NADPH oxidase; NO, Nitric Oxide; O2^.-^, Superoxide; ONOO^.-^, Peroxynitrite

During systemic inflammation, the production of free radicals damages cells and the organism, leading to significant morbidity and mortality. This process is complex and mediated by various pathways. One key molecule believed to be harmful is the reactive oxygen species Peroxynitrite (ONOO^.-^), which is the product of the reaction of nitric oxide (NO) and superoxide (O2^∙−^) [1]. Peroxynitrite may be detoxified by reacting with the amino acid Tyrosine (Fig. 1) – in turn producing 3-Nitrotyrosine (Tyr-NO2) [2–4]. Tyr-NO2 can be measured as a marker of nitrosative and oxidative stress, and has been found to be increased in different inflammatory conditions, e.g. Tyr-NO2 levels were found to be higher in smokers with COPD compared to smokers without COPD [2, 5–9]. More profound knowledge about oxidative pathways in patients with systemic infections is interesting as it is modifiable by drugs, and is thus a potential therapeutic target.

However, the significance and prognostic value of metabolomic markers of oxidative stress remains understudied. In 2013, a single-center study found the ratio of Tyr-NO2 to Tyrosine, to be predictive of mortality in critically ill patients with acute kidney injury [10]. To the best of our knowledge, there are no published data for other clinical patient populations. More profound knowledge about oxidative pathways in patients with systemic infections is interesting as it is modifiable by drugs, and is thus a potential therapeutic target. Herein, we measured baseline levels of Tyrosine and Tyr-NO2 in a well-defined cohort of patients with community acquired pneumonia (CAP) followed prospectively for 6 years. Our aim was to study associations of initial Tyrosine, Tyr-NO2 levels and their ratio respectively, with short-term adverse outcomes defined as death or ICU admission within 30 days, and long-term mortality. We hypothesized that a more efficient reduction in oxidative stress mirrored by an increase in Tyr-NO2, and a lowering of Tyrosine and the ratio of Tyrosine to Tyr-NO2 would be associated with better clinical outcomes.

## Methods

### Study design, inclusion and exclusion criteria

**Fig. 2:**
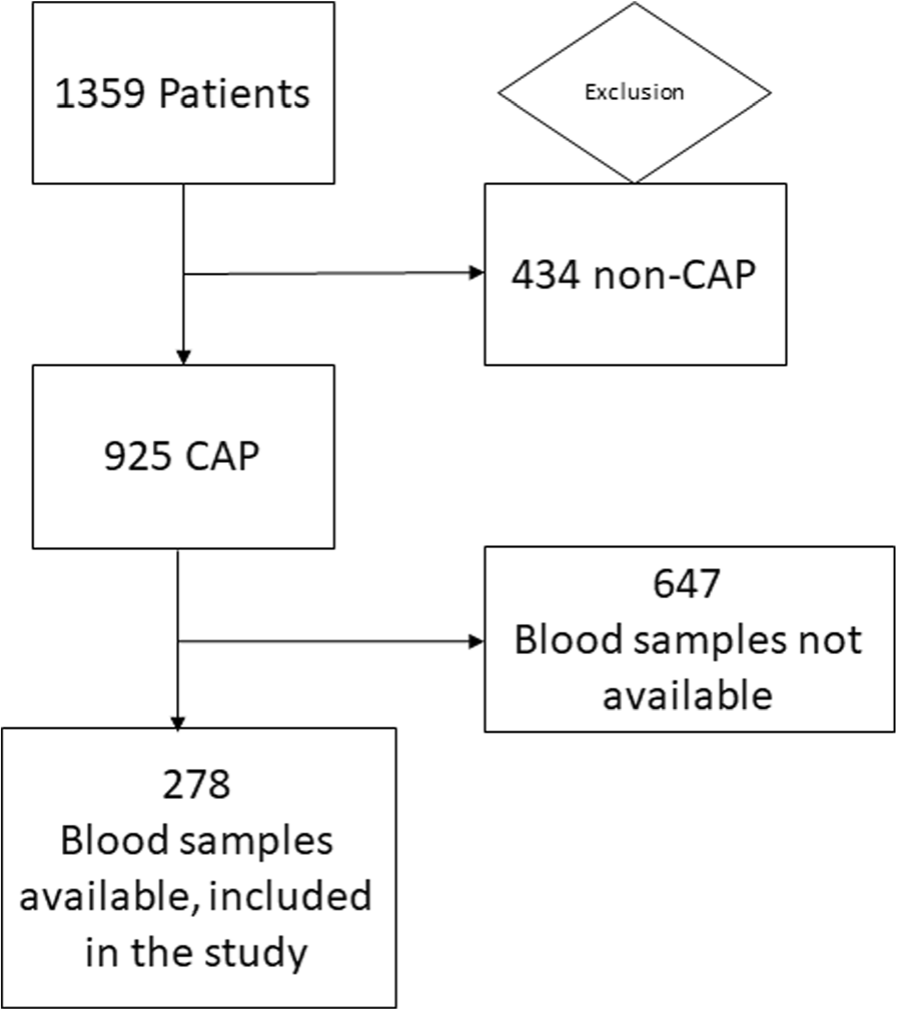
Flowchart of patient inclusion

This is a secondary analysis of a previous randomized Swiss multicenter trial with a long-term follow-up of patients over 6 years (clinicaltrials.gov identifier NCT 00350987). A total of 278 patients with a final diagnosis of CAP and available blood specimens were included (Fig. 2) [11, 12]. The initial trial included 1359 patients, of which 925 (68%) had a diagnosis of CAP with an all-cause mortality of 45% over a follow-up period of 6 years [13]. Blood specimen were taken from a random sample of patients from all participating centers where left overs were available. The initial trial was performed between October 2006 and March 2008 in six Swiss secondary and tertiary care centers [11]. The initial trial was a randomized controlled noninferiority trial, to compare strategies to improve the use of antibiotics with and without measurement of procalcitonin (PCT) in patients with lower respiratory tract infections.

Detailed information about the initial trial has been published previously [11, 12]. In brief, patients were included if they presented from the community or a nursing home to the emergency department, and met the inclusion criteria (≥18 years of age plus one or more of the following: cough, dyspnea, pleural pain, sputum production or tachypnea; plus, one or more sign of infection or positive auscultation. All patients with CAP had a radiologically confirmed infiltrate. Exclusion criteria were language barriers or dementia precluding the ability to give written informed consent, intravenous drug use, patients with terminal conditions and patients with hospital-acquired pneumonia. The blood samples and clinical data were gathered at admission to the emergency department. All involved clinicians were blinded for the results of the blood markers.

#### Analysis of serum biomarkers

At emergency department admission, blood samples from each patient were taken, centrifuged and frozen at − 80 °C directly for later measurement of metabolomic biomarkers [14–17]. Baseline Tyrosine and Tyr-NO2 were measured in blood samples of 278 patients. The *AbsoluteIDQ* p180 Kit (BIOCRATES Life Sciences AG, Innsbruck, Austria) was used for laboratory testing [18–23], after validation of the method in our lab Samples were prepared according to the manufacturer’s protocol. Analysis was performed using an Ultimate 3000 UHPLC system (Thermo Fisher, San Jose, California, USA) coupled to an ABSciex 5500 QTRAP quadrupole mass spectrometer (ABSciex, Darmstadt, Germany). Analytes were separated on a Thermo Syncronis aQ 50 × 2.1 mm 1.7 μm column prior to targeted screening using multiple reaction monitoring (MRM). Quantification of analytes was done by reference corrected to appropriate internal standards. The lowest calibrator for Tyr and NO2-Tyr was set as the lower limit of quantification (LLoQ) and not investigated in detail. Also the limit of detection was not investigated in detail. LLoQ was 5 μmol/L for Tyr and 1 μmol/L for NO2-Tyr, respectively. Calibration curve was controlled by quality control samples. Concentration of calibrator and quality control (QC) samples were given by the manufacturer and were not changed. All QC samples met the criteria adapted from the manufacturer of the p180 kit. Concentrations are reported in μmol/L [23].

#### Main outcome measurements

The primary endpoint was adverse outcome defined as a composite of ICU admission and/or death within 30 days after study inclusion. The decision for ICU admission was based on clinical judgement of the treating physician team. The secondary endpoint was death at 6 years. Trained medical students ascertained vital status through structured phone interviews at day 30, 180 and 540 and at 6 years [23, 24]. We contacted the treating general practitioners if neither patients nor their household members were reached.

#### Statistical analyses

We used STATA 12.1 (Stata Corp, College Station, TX, USA) for all statistical analyses. A *p*-value of < 0.05 was considered to indicate statistical significance. Continuous variables are reported as medians (interquartile range (IQR)) and categorical variables are expressed as percentages (numbers). We used Chi-square (Wald) tests for frequency comparisons, and we executed nonparametric (Spearman’s rank correlation) tests for two-group comparisons.

The distribution of Tyrosine and Tyr-NO2 (subsequently referred to as “biomarkers”) were skewed. After logarithmic transformation at a base of 10, the distribution of the biomarkers approximated a normal distribution. We studied associations of biomarkers with the primary endpoint by calculation of logistic regression models. We used univariate and multivariate linear regression models adjusted for predefined confounders such as age, sex, BMI and comorbidities. Odds ratios (OR) were calculated and reported with 95% CIs. Area under the receiver operating characteristic curves (AUCs) with 95% CIs are presented to illustrate discrimination. We additionally performed univariate and multivariate Cox regression models to investigate associations between biomarker levels at baseline and long-term mortality. These associations are reported as hazard ratios (HRs) with 95% confidence intervals (CIs) and significance levels for the chi-square (Wald) test. We further illustrated mortality with a Kaplan-Meier curve, stratifying patients based on the median Tyrosine level.

## Results

### Patient characteristics

Baseline characteristics of the entire cohort (*n* = 278) as well as for patients stratified by the primary endpoint (combined adverse outcome within 30 days) are presented in Table 1. The median age of patients was 71.5 years and 40.6% were female. The rate of comorbidities was high with 26.3% having a past medical history of chronic obstructive pulmonary disease (COPD), 24.1% for chronic kidney disease and 18.7% for coronary artery disease (CAD).

**Table 1:** Baseline characteristics stratified by the first endpoint (adverse outcome)

Factor	Level	Total	Non-adverse outcome at 30d	Adverse outcome at 30d	*p*-value
*N*		278	249 (89.6%)	29 (10.4%)	
Demographics					
Age in years median (IQR)		71.5 (57, 82)	71 (57, 82)	76 (67, 83)	0.078
Gender, *n* (%)	Male	165 (59.4%)	147 (59.0%)	18 (62.1%)	0.75
	Female	113 (40.6%)	102 (41.0%)	11 (37.9%)	
BMI, median (IQR)		24.8 (22.0, 27.7)	24.6 (21.8, 27.5)	26.6 (24.4, 29.5)	0.053
Comorbidities					
Coronary Heart Disease, *n* (%)		52 (18.7%)	44 (17.7%)	8 (27.6%)	0.2
Congestive Heart Failure, *n* (%)		35 (12.6%)	31 (12.4%)	4 (13.8%)	0.84
Chronic Kidney Disease, *n* (%)		67 (24.1%)	51 (20.5%)	16 (55.2%)	**< 0.001**
Diabetes mellitus, *n* (%)		41 (14.7%)	36 (14.5%)	5 (17.2%)	0.69
Tumor, *n* (%)		33 (11.9%)	27 (10.8%)	6 (20.7%)	0.12
COPD, *n* (%)		73 (26.3%)	61 (24.5%)	12 (41.4%)	0.051
Vital signs					
Pulse rate, bpm, median (IQR)		94 (82, 107)	94 (82.5, 106)	97 (75, 114)	0.75
Temperature, °C, median (IQR)		38 (37.2, 38.9)	38 (37.2, 39)	37.4 (36.7, 38.4)	**0.014**
Systolic BP, mmHg, median (IQR)		130 (118, 146)	131 (120, 147)	124 (102, 139)	**0.044**
Clinical scores					
PSI-Score	1, 2	73 (26.3%)	70 (28.1%)	3 (10.3%)	**< 0.001**
	3	56 (20.1%)	55 (22.1%)	1 (3.4%)	
	4, 5	149 (53.6%)	124 (49.8%)	25 (86.2%)	
CURB-65-Score	0, 1	125 (45.0%)	120 (48.2%)	5 (17.2%)	**< 0.001**
	2, 3	139 (50.0%)	123 (49.4%)	16 (55.2%)	
	4, 5	14 (5.0%)	6 (2.4%)	8 (27.6%)	
Markers of oxidative stress and inflammation					
Tyrosine, median (IQR)		81.7 (63.2, 118.0)	81.6 (61.8, 117.0)	84.0 (66.4, 122.0)	0.33
Tyr-NO2, median (IQR)		1.5 (0.8, 11.7)	1.5 (0.9, 12.3)	1.4 (0.5, 4.0)	0.29
Ratio Tyr-NO2/Tyrosine, median (IQR)		2.0 (1.0, 7.6)	2.1 (1.0, 8.3)	1.9 (0.9, 3.5)	0.19
CRP, median (IQR)		136 (63, 252)	132 (61, 246)	176 (106, 336)	**0.032**
PCT, median (IQR)		0.45 (0.16, 3.20)	0.40 (0.15, 2.96)	0.64 (0.21, 6.30)	0.13

Bolded *p*-values are statistically significant at *p* < 0.05. BMI, Body Mass Index; COPD, chronic obstructive pulmonary disease; bpm, beats per minute; BP, blood pressure; PSI Pneumonia Severity Index; CURB-65, score of confusion, urea, respiratory rate, blood pressure, age; CRP, initial C-reactive Protein level; PCT, initial Procalcitonin level; WBC, initial leukocyte count

### Association of markers of oxidative stress with clinical parameters and markers of inflammation

In a first exploratory analysis, we investigated correlations of clinical data at baseline, including different markers of inflammation, with the markers of oxidative stress in Spearman’s rank correlations (Table 2). We found several modest correlations of Tyrosine levels with age (*r* = 0.1748, *p* = 0.0230), and a negative correlation with levels of CRP (*r* = − 0.2662, *p* = 0.0001) and PCT (*r* = − 0.1607, *p* = 0.0207). Tyr-NO2 was correlated with initial body temperature (*r* = 0.2403, *p* = 0.0007). There were no significant associations with comorbidities and clinical risk scores. However, if adjusted for multiple testing these associations failed to persist.

**Table 2:** Rank-sum correlations of markers of oxidative stress levels with demographics, comorbidities, vital signs, clinical scores and inflammatory biomarkers

	Tyrosine	Tyr-NO2	Tyrosine/Tyr-NO2 ratio
Demographics			
Age	**r 0.1748,** ***p*** **= 0.0230**	r 0.0856, *p* = 0.2684	r 0.0158, *p* = 0.8383
Gender	r − 0.0253, *p* = 0.7441	r 0.0458, *p* = 0.5545	r 0.0508, *p* = 0.5116
BMI	r − 0.0870, *p* = 0.2608	r − 0.0100, *p* = 0.8973	r 0.0039, *p* = 0.9594
Comorbities			
Coronary heart disease	r 0.0263, *p* = 0.7059	r − 0.0020, *p* = 0.9775	r − 0.0224, *p* = 0.7483
Chronic heart failure	r 0.0586, *p* = 0.4005	r 0.1123, *p* = 0.1064	r 0.0681, *p* = 0.3285
Chronic Kidney Disease	r 0.0178, *p* = 0.7981	r 0.0104, *p* = 0.8809	r 0.0083, *p* = 0.9050
Diabetes mellitus	r − 0.0156, *p* = 0.8225	r 0.1052, *p* = 0.1305	r 0.1205, *p* = 0.0830
COPD	r 0.0430, *p* = 0.5378	r − 0.0559, *p* = 0.4228	r − 0.0832, *p* = 0.2321
Tumor	r 0.0816, *p* = 0.2413	r 0.0392, *p* = 0.5741	r 0.0149, *p* = 0.8310
Vital signs			
Heart rate	r − 0.0761, *p* = 0.2893	r − 0.0981, *p* = 0.1714	r − 0.0592, *p* = 0.4096
Systolic BP	r-0.0305, *p* = 0.6716	r − 0.0113, *p* = 0.8753	r 0.0066, *p* = 0.9270
Temperature	r − 0.0226, *p* = 0.7532	**r 0.2403,** ***p*** **= 0.0007**	**r 0.2804,** ***p*** **= 0.0001**
Clinical scores			
PSI	r 0.1264, *p* = 0.0689	r 0.0932, *p* = 0.1805	r 0.0464, *p* = 0.5060
CURB-65	r 0.1243, *p* = 0.0737	r 0.1008, *p* = 0.1474	r 0.0508, *p* = 0.4662
qSOFA	r 0.0307, *p* = 0.6594	r 0.1132, *p* = 0.1034	r 0.1074, *p* = 0.1226
Biomarkers			
CRP	**r − 0.2662,** ***p*** **= 0.0001**	r − 0.0994, *p* = 0.1540	r − 0.0338, *p* = 0.6286
PCT	**r − 0.1607,** ***p*** **= 0.0207**	r 0.0519, *p* = 0.4579	r 0.1158, *p* = 0.0967
WBC	r − 0.0586, *p* = 0.4018	r 0.0080, *p* = 0.9091	r 0.0397, *p* = 0.5703

Bolded *p* values are statistically significant at *p* < 0.05. BMI, Body Mass Index; COPD, chronic obstructive pulmonary disease; BP, blood pressure; PSI Pneumonia Severity Index; CURB-65, score of confusion, urea, respiratory rate, blood pressure, age; CRP, initial C-reactive Protein level; PCT, initial Procalcitonin level; WBC, initial leukocyte count

### Association between markers of oxidative stress and short-term adverse outcome

After 30 days of study inclusion, the composite primary endpoint was reached by 29 (10.4%) of patients. Lower levels of Tyr-NO2 and the ratio of Tyr-NO2 to Tyrosine were associated with a lower risk for 30-day adverse outcome, as evidenced by a PSI, BMI and comorbidities-adjusted OR of 0.44 (95% CI 0.20 to 0.95, *p* = 0.039) and 0.98 (95% CI 0.98 to 0.99, *p* = 0.043) respectively. Results for Tyrosine were not significant in univariate analysis (OR 3.3, 95% CI 0.63 to 17.36), *p* = 0.159), nor in multivariate analysis (OR 0.84 (95% CI 0.15 to 4.65.), *p* = 0.841).

To assess discrimination, we further calculated AUCs. Overall, these markers of oxidative stress showed poor prognostic power with an AUC for Tyrosine of 0.55 (95% CI 0.44 to 0.66) for Tyr-NO2 of 0.43 (95% CI 0.31 to 0.55) and for the ratio of Tyr-NO2 to Tyrosine of 0.42 (95% CI 0.31 to 0.53) (Table 3).

**Table 3:** Association of initial levels of oxidative stress markers with endpoints

Entire Cohort (*n* = 278)	Adverse outcome at 30d	6 year Mortality
	OR (95% CI); *p*-value	HR (95% CI); *p*-value
Tyrosine^a^		
Univariate	3.30 (0.63 to 17.36), *p* = 0.159	1.88 (0.90 to 3.91), *p* = 0.093
Multivariate^b^	0.84 (0.15 to 4.65), *p* = 0.841	1.58 (0.72 to 3.47), *p* = 0.256
AUROC	0.55 (0.44 to 0.66)	0.60 (0.54 to 0.67)
Tyr-NO2^a^		
Univariate	0.65 (0.35 to 1.20), *p* = 0.167	0.96 (0.73 to 1.27), *p* = 0.778
Multivariate^b^	0.44 (0.20 to 0.96), *p* = 0.039	0.81 (0.6 to 1.1), *p* = 0.181
AUROC	0.43 (0.31 to 0.56)	0.50 (0.42 to 0.58)
Ratio Tyr-NO2/Tyrosine		
Univariate	0.91 (0.81 to 1.02), *p* = 0.098	0.99 (0.95 to 1.02), *p* = 0.457
Multivariate^b^	0.98 (0.97 to 0.99), *p* = 0.043	1.00 (0.99 to 1.00), *p* = 0.216
AUROC	0.42 (0.30 to 0.53)	0.48 (0.40 to 0.56)

^a^Values were log-transformed; ^b^ Multivariate analysis adjusted for PSI-Class (≤3 vs ≥4) BMI and comorbidities

### Association between markers of oxidative stress and long-term mortality

**Fig. 3:**
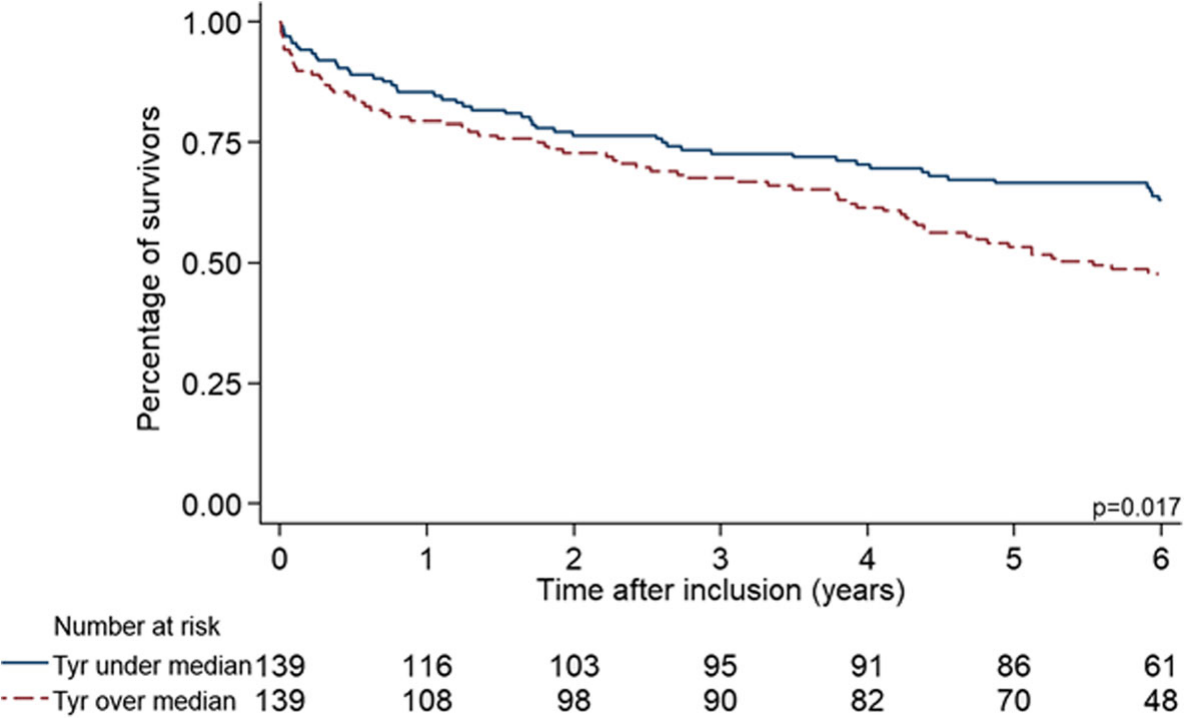
Kaplan-Meier survival estimates for Tyrosine. Six-year survival estimates, unadjusted, stratified based on the median Tyrosine level

Mortality at day 30 was 5% (*n* = 14) and increased to 17.3% (*n* = 48) after one year and 45% (*n* = 125) after five years of follow-up. Higher Tyr-NO2 levels (adjusted hazard ratio 0.81, 95% CI 0.60 to 1.1, *p* = 0.181) showed a trend towards improved survival, but this result did not reach statistical significance. These results are also graphically displayed in a Kaplan Meier plot stratified by the median Tyrosine levels (Fig. 3).

## Discussion

Oxidative stress has been recognized as a potentially modifiable risk factor in inflammation and infection, but there is a lack of clinical data regarding the significance of specific metabolomic markers of oxidative stress. In this study of CAP patients followed prospectively for 6 years, we tested the hypothesis that a more efficient reduction in oxidative stress, mirrored by metabolomic markers of oxidative stress, would be associated with better health outcomes. Our results indicate that levels of Tyr-NO2 tend to be lower and the ratio of Tyrosine to Tyr-NO2 tends to be higher in patients with adverse short-term outcomes and higher long-term mortality, however many results did not reach levels of statistical significance.

In the setting of a systemic inflammatory disease such as CAP, an increase in the production of ONOO^.-^ takes place, which is primarily mediated by an upregulation of inducible nitric oxide synthase in macrophages, leading to nitric oxide reacting with also increased levels of superoxide O2^.-^ [25]. This process represents the generation of nitrosative stress in addition to oxidative stress, which may lead to uncontrolled nitrosylation of molecules and in turn to dysfunction of proteins, cells and organs [26]. It is important to bear in mind, that the equilibrium between Tyrosine, Tyr-NO2 and Peroxynitrite is quite complex and can be influenced by a plethora of interfering physiological processes, such as alternative elimination pathways of NO via the reaction with metal ions, principally with iron [27], the not well known pathway of catabolism of Tyr-NO2 [28]. We hypothesized that the capacity to process oxidative stress via this pathway could be indicative of the ability to resist damage from oxidative insults. While knowledge of these pathways is mainly based on preclinical studies [1–9], there is a lack of patient data showing markers of oxidative stress is associated with clinical outcomes [29–31]. Herein, we measured initial levels of Tyrosine and Tyr-NO2 levels in a relatively large and well-defined cohort of CAP patients over a 6-year follow-up period. Interestingly, in an analysis adjusted for age, sex and comorbidities, low admission levels of Tyr-NO2 were associated with a lower risk for reaching the primary composite endpoint and showed a trend with the secondary endpoint of death at 6 years, however this association does not persist after adjustment for multiple testing. Although levels of Tyrosine and the Tyr-NO2/Tyrosine ratio did not show a significant correlation with either the composite endpoint or with 6-year mortality, trends persisted indicating worse outcomes in patients with higher Tyrosine levels and lower ratios. Importantly, from our analysis it remains unclear whether systemic inflammation impedes the Tyrosine/Tyr-NO2-pathway, implying an observational correlation; or whether a higher capacity for detoxification is a patient-specific characteristic (measure of resources), suggesting a causative relation of Tyrosine nitrosylation on clinical outcome. Yet, we did not find associations of comorbidities and levels of Tyrosine, Tyr-NO2 or their ratio. Blood markers of inflammation (i.e. CRP and PCT), on the other hand, showed a weak, however significant association with Tyrosine, as well as age. However, these associations did not persist after adjusting for multiple testing and should thus be rather viewed as exploratory. A higher Tyr-NO2 and Tyr-NO2/Tyrosine ratio were associated with body temperature, possibly reflecting a more intact physiological response to inflammation.

The main strengths of this study are the measurement of different oxidative stress markers in a well characterized cohort of CAP patients where clinical data have been sparse on the significance of oxidative stress markers. Our cohort included patients with CAP of various severities, who were followed over 6 years. Still, there are a number of limitations to this study. Firstly, the number of included patients reaching an endpoint was relatively small, limiting the statistical power. In fact, a post-hoc power analysis showed that we only had 20% and 34% power to find a significant difference in levels of Tyrosine and Tyr-NO2, respectively, regarding the primary endpoint. This may explain the borderline significant results found in some of the analyses. Secondly, we did not measure the serum levels of Peroxynitrite in the original blood samples, which could have given a more complete picture of the biochemical pathway. Thirdly the data were generated in Swiss hospitals only, limiting generalizability to other countries. Further, the cause of death was not verified by autopsy. Because of this, we investigated all-cause mortality rather than mortality related to infection. We did not differentiate the degree of severity of infection (e.g. whether sepsis or septic shock was present on presentation). Finally, we did not further explore whether oxidative stress markers would improve risk scores of pneumonia such as CURB65 because our results indicated a rather low discriminative performance in regard to AUCs. We also did not measure the maximum time to analysis for the obtained blood samples. As a secondary analysis of a randomized controlled trial, this investigation is rather hypothesis-generating, but may help to stimulate more interest into oxidative stress in future studies. Study of an animal model might help to further advance the understanding of the Tyrosine/Tyr-NO2 pathway, as well as the effects of therapeutic intervention on outcomes.

## Conclusion

In conclusion, our data suggest that more efficient reduction in oxidative stress, mirrored by metabolomic markers, could be associated with fewer adverse clinical outcomes in CAP patients at short-term, but not at long-term. Further research, however, is needed to validate our findings and to better understand causal effects. Also, whether therapeutic modulation of the Tyrosine/Tyr-NO2 pathway improves clinical outcomes should be evaluated in future studies.
